# An experimentally validated fading model for THz wireless systems

**DOI:** 10.1038/s41598-021-98065-x

**Published:** 2021-09-21

**Authors:** Evangelos N. Papasotiriou, Alexandros-Apostolos A. Boulogeorgos, Katsuyuki Haneda, Mar Francis de Guzman, Angeliki Alexiou

**Affiliations:** 1 Department of digital systems, University of Piraeus research center, 18534 Piraeus, Greece; 2 School of Electrical and Engineering, Aalto University, Ghent, Finland

**Keywords:** Engineering, Electrical and electronic engineering, Mathematics and computing, Statistics

## Abstract

As the wireless world moves towards the sixth generation (6G) era, the demand of supporting bandwidth-hungry applications in ultra-dense deployments becomes more and more imperative. Driven by this requirement, both the research and development communities have turned their attention into the terahertz (THz) band, where more than $20\,{\text {GHz}}$ of contiguous bandwidth can be exploited. As a result, novel wireless system and network architectures have been reported promising excellence in terms of reliability, massive connectivity, and data-rates. To assess their feasibility and efficiency, it is necessary to develop stochastic channel models that account for the small-scale fading. However, to the best of our knowledge, only initial steps have been so far performed. Motivated by this, this contribution is devoted to take a new look to fading in THz wireless systems, based on three sets of experimental measurements. In more detail, measurements, which have been conducted in a shopping mall, an airport check-in area, and an entrance hall of a university towards different time periods, are used to accurately model the fading distribution. Interestingly, our analysis shows that conventional distributions, such as Rayleigh, Rice, and Nakagami-m, lack fitting accuracy, whereas, the more general, yet tractable, $\alpha$–$\mu$ distribution has an almost-excellent fit. In order to quantify their fitting efficiency, we used two well-defined and widely-accepted tests, namely the Kolmogorov–Smirnov and the Kullback–Leibler tests. By accurately modeling the THz wireless channel, this work creates the fundamental tools of developing the theoretical and optimization frameworks for such systems and networks.

## Introduction

The teraherthz (THz) wireless communications have been identified as a promising enabler for the sixth generation (6G) wireless technologies, because the THz band offers a contiguous bandwidth of more than ${20\,{\text {GHz}}}$^1,2^. The THz band (0.1–10 THz) is envisioned to be utilized in the deployment of both indoor and outdoor wireless systems. In the recent years, both academics and industry have focused their attention on the development of outdoor and especially indoor THz wireless systems^2–4^. In more detail, regarding the indoor THz wireless communications significant standardization bodies are in the process of publishing spectrum allocation regulations and standards such as, the Institute of Electrical and Electronics Engineers (IEEE) standard (Std.) 802.15.3d-2017, International Telecommunication Union (ITU)-T SM. 2353 Report (2015 and 2016), European Telecommunications Standards Institute (ETSI) mWT, Federal Communications Commission (FCC): American Spectrum Regulations, and European Communications Commission (ECC): Europe Spectrum Relations^5–9^. However, despite all this effort the channel modeling of indoor THz wireless communications has not been yet adequately settled^3,4,10–24^.

Due to the severe propagation losses in the THz band, the wireless communications in this frequency range rely heavily on the line-of-sight (LoS) component of the received signal^11,12,25^. Moreover, by taking this into account, the THz channel is commonly modeled by considering only the large-scale propagation phenomena, namely the shadowing and the deterministic pathloss^11–13,19–24^. The pathloss in the THz band is expressed as the product of the free space and molecular absorption loss^11^. In order to acquire and model the pathloss coefficient of the molecular absorption loss, the use of spectroscopic databases yielding the different molecular absorption lines is needed^3^. To mitigate the need to access the spectroscopic databases, various simplified molecular absorption loss models were developed for the ranges of 100–450 GHz, 200–450 GHz and 275–400 GHz^11,19,20^. Moreover, by employing these simplified models the THz channel was assumed to consist of a single coefficient in the LoS direction, which was obtained as the product of the free-space loss and the molecular absorption loss^11,19,20^. Meanwhile, LoS and non-line-of-sight (NLoS) channel measurements for various narrowband indoor wireless communications links operating at 28 GHz and 140 GHz were performed^12,13^. In these works, based on the measured received signal power of the multipath components of the different links, the respective millimeter wave (mmWave) and THz channels were deterministically modeled as the sum (in dB) of an exponential pathloss and a lognormal shadowing distribution. The parameters of the exponential pathloss and the variance of shadowing were extracted by making use of the received signal powers of the observed links. Additionally, a single path theoretical THz channel model for nano-scale machine communications within vegetation was derived, where the receiver (RX) was assumed to detect signals only from the LoS direction^22,24^. More specifically, it was assumed that, the channel was composed by two coefficients, which were the pathloss modeled as the product of the free space and molecular absorption losses and a lognormal shadowing. Also, a new paradigm of aerially suspended nano-nodes to bridge the disjoint internet-of-things (IoT) THz networks was proposed. In this work, the proposed LoS THz channel model between the TX and the RX nano-nodes was expressed in terms of the deterministic pathloss. More specifically, the pathloss model took into account the environmental conditions, the spreading and molecular absorption losses, the transceivers distances, the angle of arrival, and the RX antenna dimensions^26^. Moreover, two single-frequency and one multiple frequency THz pathloss models were introduced. These models were extracted by employing multiple indoor wideband measurements in the range of 220–330 GHz^27^. Furthermore, an indoor channel model for mmWave and THz frequencies operating at 28 and 140 GHz was developed. This model included the cases of omni directional and directional pathloss as well as cluster channel statistics, namely their number, delays and powers^28^.

Despite the heavy rely on the existence of the LoS component in THz wireless communications, there are aerosols in the atmospheric medium, as well as objects laid in the propagation environment that can act as scatterers^12,13,29^. Hence, there can be THz multipath components with significant power capable of being detected by the RX even if they arrive from NLoS directions^4,12,21^. The existence of multipath components having different levels of received power, angles of arrival, angles of departure and delay times means that the received signal power at the RX can have deep and time varying fast fades^30^. Those phenomena are parts of the stochastic small-scale fading^30^. According to the technical literature there are works that perform and employ theoretical as well as experimental THz channel modeling by taking into account phenomena belonging to the small-scale fading^3,4,10,12–18,21,31^. It was observed by means of experimental THz channel measurements that the small-scale fading in this band can be modeled by means of Rice, Nakagami-m and Rayleigh distributions^3^. Considering this, the more generic $\alpha$–$\mu$ distribution was employed to model the small-scale fading of a THz backhaul wireless system and the performance was evaluated under different levels of transceiver antennas misalignment, hardware impairements and fading severity, in terms of outage probability and ergodic capacity^3,10^. Meanwhile, a stochastic two dimensional geometrical channel model for indoor THz communications was developed. By employing this model, a parametric multipath Rice fading model for THz communications was elaborated^16,17^. Furthermore, a stochastic indoor THz channel model was introduced, were the small-scale fading attenuation factor was obtained by a Rayleigh or Nakagami-m disttribution under NLoS conditions and as a Rice or Nakagami-m in LoS. The aforementioned claims were validated by means of experimental measurements, which took place inside an anechoic chamber^15^. In the meantime, experimental received signal power measurements of multiple LoS and NLoS transceiver links recorded in a shopping mall were employed to derive the suitable small-scale fading distribution for THz systems operating at 140 GHz. Then, based on those measurements it was concluded that for the LoS and NLoS links the distribution that most accurately describes the measured channel data is the Weibull and Nakagami-m, respectively. To support this claim the fitting of those theoretical fading distributions to the empirical data was evaluated in terms of the goodness of fit Kolmogorov–Smirnov (KS) test^18^. Also, another approach to model indoor THz wireless systems operating at the range of 240–300 GHz was by assuming a small-scale fading distribution expressed as a sum of individual Gamma distributions. The suitability of the Gamma distribution for THz channel modeling was verified by means of the KS test, the Kullback Leibler (KL) divergence test and the weighted relative mean difference error metric, which tested the fitting of the analytical expression to the measured data^4^. Furthermore, a measurement based channel model for LoS and NLoS conditions was proposed for THz transceivers operating in the range 126–156 GHz and it was based on the extended Saleh–Valenzuela channel model. In this model the large-scale fading was expressed in terms of exponential pathloss and shadowing, while the small-scale fading amplitude was obtained by a novel distribution. The accuracy of the model was evaluated by means of indoor exprerimental measurements^21^. Meamwhile, the small-scale fading of a holographic multiple-input–multiple-output (MIMO) system suitable for mmWave and THz communications was theoretically proposed. There, the small-scale fading was modeled as a zero-mean, spatially stationary, and corelated Gaussian scalar random field^31^. Also, quite recently the fluctuating two-ray (FTR) model has been considered as a promising candidate to accurately model the small-scale fading statistics of THz wireless channels. In more detail, a THz measurement campaign at 300.4 GHz was conducted in a train facility test center, where various obstacles were present, such as trains, tracks and lampposts^32^. Then, the small-scale fading statistics of those measurements were verified to be very accurately fitted by the FTR fading model, which performed significantly better than the Rice, Gaussian and Nakagami-m distributions^33^. Finally, there are publications in lower frequency bands, such as the mmWave band, which study the small-scale fading distributions of the wireless channels by means of the $\alpha$–$\mu$ and $\kappa$–$\mu$ distributions^34–36^. However, due to the fact that the notion of scatterer and blocker are dependent on the wavelength, while moving to higher frequencies such as those of the THz band; the need to re-investigate those terms arises^25,37^.

To the best of the authors knowledge, no fading distribution modeling the channel of indoor THz systems has been yet documented to be based not only on measurements conducted in multiple environments, but also on different time periods. Motivated by this, in this work the measurements of LoS and NLoS links of three indoor measurement environments were exploited. In more detail, it was made use of the measurements conducted within the premises of a shopping mall, an airport check in area and the entrance hall of Aalto university in Finland^12,13^. The shopping mall is located at Espoo Finland and the airport is in Helnsiki. In both of these scenarios the measurements were conducted in November 2016, meanwhile the measurements in the university entrance hall were performed in the time period from January to March 2021. For each link multiple channel gain measurements were recorded, which were then used in this work to perform small-scale fading statistics. As will be presented in the “Method” section, first the measurements of each link will be preprocessed to obtain the channel gain of the recorded multipath components. Then, to increase the number of the different channel realizations in each link, a method based on adding random phases to the path amplitudes will be employed. By making use of the resulting channel realizations of each link, the empirical probability density function (PDF) and cummulative density function (CDF) are fitted to the analytical distributions of $\alpha$–$\mu$, Nakagami-m, Rayleigh, Rice ,and lognormal^30,38,39^. The parameters of the analytical distributions are obtained by fitting them to the empirical ones of the channel gain. This is accomplished by means of non linear regression machine learning. The accuracy of the fit of the analytical distributions to the corresponding empirical ones is validated by means of the KS goodness of fit test and KL divergence test^40,41^. All the analytical distributions except Rayleigh passed the KS test. Moreover, it is observed that the $\alpha$–$\mu$ distribution yields almost a perfect fit for all the links of all the examined scenarios, which is not the case for Rice, Nakagami-m and lognormal. To validate this observation the KL test is employed. According to this test, $\alpha$–$\mu$ results to the least distance from the empirical PDF. Moreover, it should be noted that the aim of this work is not only to identify a fading distribution capable of accurately modeling the small-scale fading statistics of THz wireless channels, but also to be analytically tractable. The PDF of the FTR fading distribution is expressed as a series of a Legendre polynomial that contains the confluent hypergeometric function^42^, Eq. (13)]. Also, the definition of the PDF expression of FTR increases the complexity to find the distribution parameters needed to perform fitting to the THz channel data. Furthermore, the THz channel modeling by means of sum of *N* independent Gamma distributions has been found to yield very accurate fit to THz channel measurements^4^. However, to perform fitting to the channel measurements one should identify the suitable parameters for each of the *N* different Gamma distributions, which increases detrimentally the complexity of this process^4^, Eq. (8)].

## Results

This section focuses on the presentation of the fitting results of $\alpha$–$\mu$, Nakagami-m, Rice and lognormal distributions to the empirical channel gain distributions of the links. In more detail, first a short presentation of the measurement setup and sites takes place. Then, it is followed by the statement of the superior fitting of $\alpha$–$\mu$ to the empirical data when compared to Rice, Rayleigh, Nakagami-m and lognormal. Subsequently, some indicative figures illustrating the fit of $\alpha$–$\mu$ to the empirical channel gain PDF and CDF to LoS and NLoS links of the three scenarios is presented.

### Measurement setup and sites

**Figure 1:**
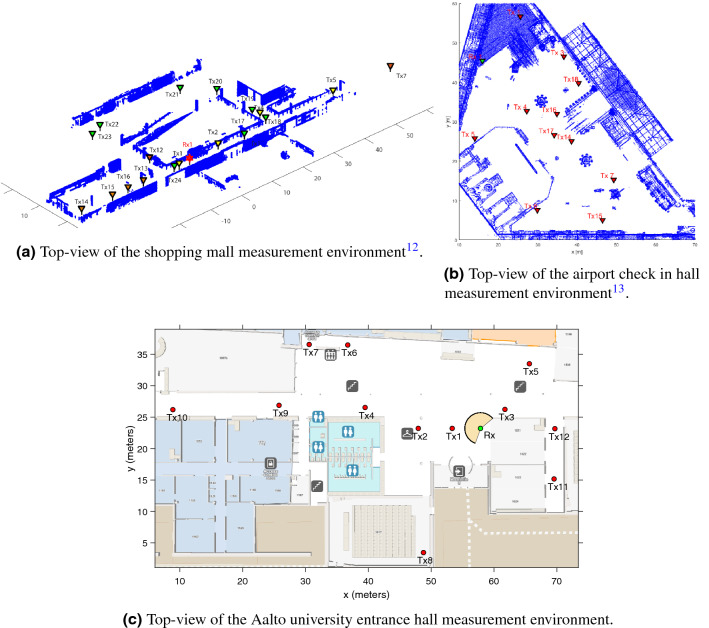
Measurement setups.

In both the shopping mall and airport check in measurement scenarios the transmissions were conducted at the center frequency of ${143.1 \,\text {GHz}}$ with a total bandwidth of ${4 \,\text {GHz}}$, and the RX antenna was rotated with an angular step of ${5^{\circ }}$^12,13^. Furthermore the transmitter (TX) was equipped with an omni-direction antenna yielding a gain of ${0 \,\text {dBi}}$, whereas the RX was equipped with a directional horn antenna achieving a gain of ${19 \,\text {dBi}}$. During the measurement campaign in the shopping mall the paths of 18 independent TX–RX links were measured. More specifically the RX for all the experiments was placed at the same position, while the TX was also static but placed at 18 different positions each one corresponding to a different TX–RX link. Additionally, the experiments were conducted at a time of the day that no people were at the premises, hence the measured paths are not impaired by human blockage. The only blockers that could interrupt the LoS link were a pillar and an escalator. From the total of 18 measured links, only three were in NLoS conditions, namely the links 18, 20 and 22. Furthermore, Fig. 1a, illustrates the top-view of the shopping mall floor in which the measurements were conducted. Figure 1b, illustrates the top-view of the airport check in hall. During the measurement campaign 11 independent TX–RX links were measured^13^. In all the experiments the RX was placed at the same position. Meanwhile, the TX was also static but placed at 11 different positions, where each position corresponds to a different TX–RX link. The measurements took place at a time of the day that no people were at the premises, hence the measured paths are not impaired by human blockage. The only blocker present was a check in kiosk, which caused link 16 to be in NLoS.

The same channel sounder setup elaborated for the shopping mall and airport was utilized except for some key differences such as the center radio frequency (RF) is 142 GHz, RF power to antenna is 5 dBm, TX and RX antenna heights are 1.85 m and angular step is $10^{\circ }$. The entrance hall map and antenna locations are illustrated in Fig. 1c. Note that there is a cylindrical building pillar and plants obstructing the LoS path of links 4 and 6, respectively. Hence, among the 12 TX–RX links considered, only ${\text {TX}}_1$ and ${\text {TX}}_2$ have LoS path to the RX. Measurements were also performed while the antennas are stationary and there are no moving objects in the whole entrance hall. Furthermore, for the links 1, 5, 6, 7, 9, 10 and 12 several repeated measurements were recorded, in order to investigate the repeatability of the channel characteristics.

The channel gain estimates of each path, from the channel sounding of all the scenarios, have at least ${10\,{\text {dB}}}$ signal-to-noise-ration (SNR). The noise observed to the measured received power of each link is attributed to the vector network analyzer of the employed receiver. Despite this fact, many LoS and NLoS paths of the employed measurements, have SNR greater than ${30\,{\text {dB}}}$. Therefore, the effect of noise is minimal in the fading statistics studies performed in this work. Generally, it should be noted that, the effect of noise must be kept minimal when performing channel modeling studies. Moreover, the measurement method used to obtain the empirical channel data, allows spatio-temporal sounding, i.e., to see fading of channel coefficients over space and frequency. Those fading coefficients are what systems in realistic scenarios obtain.

### The observed advantage of $\alpha$–$\mu$ fitting over well known distributions in THz channel modeling

**Figure 2:**
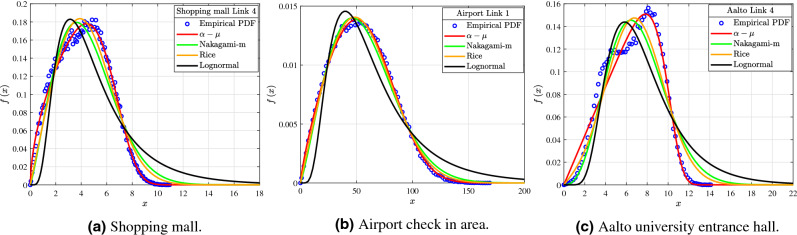
Fitting of $\alpha$–$\mu$, Rice, Nakagami-m and Lognormal PDF to the empirical ones.

In wireless communications were the LoS paths are the dominant contributors to the received power, the small-scale fading is commonly modeled by a Rice, Nakagami-m, Rayleigh or lognormal distribution^15–18^. In this work the channel gain measurements of the three scenarios are fitted by Rice, Nakagami-m, $\alpha$–$\mu$, Rayleigh and lognormal fading distributions. The fitting of those distributions to the empirical ones of the data is evaluated by the goodness of fit KS and the KL divergence test. In Tables 1, 2, 3, 4, 5 and 6 the KL test of the $\alpha$–$\mu$, Rice, Nakagami-m and lognormal distributions is represented as ${{\text {KL}}_{\alpha{\text{--}}\mu }}$, ${{\text {KL}}_R}$, ${{\text {KL}}_N}$ and $KL_L$, respectively. Additionally, the TX and d columns stand for the index of the transmitter antenna and the TX–RX distance, respectively. The columns $\alpha$, $\mu$ and $\beta$ represent the $\alpha$–$\mu$ distribution parameters. Meanwhile, the columns *K* and $\Omega _K$ represent the Rice distribution parameters, whereas the columns *m* and $\Omega _N$ are the parameters of the Nakagami-m distribution. Also, the columns $\mu _L$ and $\sigma _L$ stand for the lognormal distribution parameters. For the KS-test columns the check mark indicates that the links passed the KS-test, whereas the xmark that they did not. Moreover, for the LoS columns the check mark means that the corresponding link is in LoS conditions, whereas the xmark that it is in NLoS. Also, the number of samples of the empirical distributions CDF used in the KS test is given by the column *N*, in Tables 1, 3 and 5. From Tables 1, 2, 3, 4, 5 and 6 it is shown that all the distributions provide an adequate fit in terms of the KS test with a significance level of $5\%$. However, there are a lot of links where by observing the fit of the analytical distributions to those of the empirical ones, $\alpha$–$\mu$ achieves a significantly better fit. This finding is further strengthened by the KL test. In more detail, for the shopping mall links 4, 5, 7, 12, 14, 16, 18, 19 and 21 the KL test shows that the PDF of $\alpha$–$\mu$ has a significant less distance from the empirical PDF, when compared to the PDF of Rice and Nakagami-m. The same can be said for the link 1 of the airport measurement site and for the link 4 of the Aalto university site. For the rest of the links of the shopping mall according to the KL value it is shown that $\alpha$–$\mu$ and Rice provide a good fit to the data, while Nakagami-m has the worst performance. Meanwhile, for the rest of the airport links all the examined distributions achieve an accurate fit to the data and have similar KL values. Furthermore, for Aalto university site links 3, 5, 6, 7, 10 and 12 according to the KL results $\alpha$–$\mu$ and Rice achieve a goood fit to the data, while Nakagami-m performs worse. Meanwhile, for the links 1, 2 and 9 of this scenario based on the KL results the three distributions provide an accurate fit to the data and have similar KL values. Meanwhile, by observing the values of the KL test, the lognormal distribution by far performs the worst in comparison with the rest of the examined distributions. Furthermore, Fig. 2 illustrates some indicative fits of the analytical PDFs to the empirical ones for the shopping mall, airport and Aalto entrance hall, where $\alpha$–$\mu$ performs much better than Rice, Nakagami-m and lognormal. The blue circles stand for the empirical PDF, while the continuous red, green, orange and black lines represent the analytical PDF of $\alpha$–$\mu$, Nakagami-m, Rice and lognormal distributions, respectively. Moreover, from Fig. 2 it is observed that the tails of the analytical lognormal PDFs have severe discrepancy from their corresponding empirical PDFs. Also, from Fig. 2b it is observed that the empirical PDF of the airport scenario is moved to the left, when compared to the corresponding ones of the shopping mall and university entrance hall measurement scenarios. This is illustrated by examining the empirical PDFs of the links presented in Fig. 2a,c. This displacement of the airport check-in hall empirical PDF is caused by the existence of stronger multipath components in this environment, when compared to the other two measurement sites. Hence, the probability of observing deeper fades in the channel measurements performed in the airport, in comparison with those conducted in the shopping mall and university entrance hall is increased. In more detail, this finding can be further supported by the greater delay spread of multipath components in the airport check-in hall, as opposed to those of the shopping mall and university entrance hall scenarios^12,13^. Finally, it should be noted that, no parameters of the Rayleigh distribution are presented in Tables 1, 2, 3, 4, 5 and 6 for neither of the three measurements scenarios. This is due to the fact that no analytical Rayleigh PDF would pass the KS test.

**Table 1:** Shopping mall links parameters of the $\alpha$–$\mu$ distribution.

TX	$d\;\left( \text {m}\right)$	$\alpha$	$\mu$	$\beta$	$KL_{\alpha{\text{--}}\mu }$	KS-test	N	LoS
1	5.1	3.35467	1.11365	11.58104	0.01108	✓	124	✓
2	10.04	3.28199	1.71725	10.74124	0.00465	✓	104	✓
4	27.51	4.9364	0.31735	5.41837	0.02863	✓	111	✓
5	47.14	4.88788	0.3199	5.27926	0.0293	✓	103	✓
7	65.2	4.04712	0.44506	3.40545	0.00847	✓	71	✓
12	10.27	3.45388	0.51571	6.94184	0.00597	✓	83	✓
13	15.14	3.1958	0.79901	11.8903	0.00431	✓	94	✓
14	35.08	6.54459	0.23562	6.0124	0.01849	✓	55	✓
15	25.03	3.11872	0.91962	9.8334	0.01271	✓	113	✓
16	20.09	3.5697	0.54063	9.19752	0.00134	✓	73	✓
17	18.06	3.01129	0.69601	8.51768	0.0032	✓	101	✓
18	26.53	2.92801	0.61844	4.35616	0.02659	✓	108	✗
19	27.58	3.31085	0.67657	4.05838	0.00832	✓	90	✓
20	33.13	2.68785	0.69127	3.84872	0.01472	✓	99	✗
21	33.28	3.1831	0.59946	5.34915	0.00184	✓	63	✓
22	26.98	3.43304	1.29688	4.79929	0.01178	✓	90	✗
23	24.66	3.3977	0.96138	6.11017	0.01818	✓	124	✓
24	3.15	3.37619	2.77203	15.14883	0.00903	✓	107	✓

**Table 2:** Shopping mall links parameters of the Rice, Nakagami-m and Lognormal distributions.

TX	*K*	$\Omega _{R}$	$KL_{R}$	*m*	$\Omega _{N}$	$KL_{N}$	$\mu _L$	$\sigma _L$	$KL_L$	KS-test
1	4.12346	122.15925	0.00738	2.72371	127.42901	0.10328	2.37832	0.32266	0.83088	✓
2	7.12574	108.45571	0.00386	4.22268	111.58616	0.05866	2.328	0.25333	0.45482	✓
4	0.83431	23.23858	0.24327	1.08482	24.33919	0.41207	1.48406	0.58739	2.50743	✓
5	0.8035	22.08793	0.24938	1.07648	23.11916	0.41334	1.45735	0.59501	2.46297	✓
7	1.12911	9.62603	0.119	1.23751	10.14863	0.29989	1.06052	0.52977	2.38217	✓
12	0.86595	41.24310	0.04303	1.14194	43.17494	0.10486	1.76972	0.55775	1.05564	✓
13	2.08032	124.90146	0.0022	1.70487	132.32113	0.05578	2.3703	0.42407	0.68613	✓
14	1.01696	27.25016	0.18944	1.13075	28.72291	0.30801	1.57912	0.57736	1.48182	✓
15	2.51219	86.46958	0.00335	1.92019	91.35197	0.09136	2.19294	0.39497	1.02008	✓
16	1.20945	72.12805	0.016	1.28485	76.1611	0.06785	2.06926	0.51079	0.74626	✓
17	1.23276	63.93201	0.00716	1.30778	67.5802	0.06402	2.01097	0.50369	1.01511	✓
18	0.6556	16.89162	0.07058	1.08268	17.51565	0.14797	1.30753	0.58079	1.81433	✓
19	1.6426	14.3393	0.02606	1.49142	15.20281	0.20446	1.27905	0.46151	2.28952	✓
20	0.60182	13.47214	0.0362	1.07251	13.93943	0.09268	1.19328	0.58199	1.70689	✓
21	0.99357	24.92385	0.01956	1.20097	26.20522	0.07826	1.52779	0.53564	1.02775	✓
22	5.42769	21.25687	0.03709	3.36863	21.99173	0.154	1.50777	0.28737	1.06022	✓
23	3.39522	33.55362	0.0321	2.35782	35.1651	0.22428	1.7276	0.35083	1.79395	✓
24	13.58222	220.94597	0.01412	7.43773	224.51724	0.03227	2.68985	0.18783	0.18862	✓

**Table 3:** Airport links parameters of the $\alpha$–$\mu$ distribution.

TX	$d\;\left( \text {m}\right)$	$\alpha$	$\mu$	$\beta$	$KL_{\alpha{\text{--}}\mu }$	KS-test	*N*	LoS
1	15.36	3.00726	0.59581	65.86256	0.00045	✓	86	✓
3	21.21	2.10678	0.93887	37.01822	0.00067	✓	127	✓
4	17.46	2.03856	0.97323	38.54941	0.00061	✓	137	✓
5	20.16	2.6129	0.77404	39.97227	0.00066	✓	118	✓
6	40.77	2.8835	0.73642	35.2643	0.00106	✓	96	✓
7	45.34	2.00807	1.00254	33.37845	0.00058	✓	114	✓
14	30.8	2.05484	0.95285	35.78717	0.00048	✓	120	✓
15	50.81	2.02206	0.98845	32.82858	0.00046	✓	113	✓
16	23.53	2.0364	0.97998	39.52365	0.00079	✓	148	✗
17	26.6	2.63411	0.76323	39.51715	0.00073	✓	113	✓
18	25.48	2.18022	0.89005	37.85602	0.00076	✓	119	✓

**Table 4:** Airport links parameters of the Rice, Nakagami-m and Lognormal distributions.

TX	*K*	$\Omega _{R}$	$KL_{R}$	*m*	$\Omega _{N}$	$KL_{N}$	$\mu _L$	$\sigma _L$	$KL_L$	KS–test
1	0.66982	3839.29866	0.00217	1.08142	3989.37775	0.00587	4.02321	0.57953	0.09026	✓
3	0.25999	1340.67651	0.00064	1.01572	1356.06881	0.00091	3.47072	0.60523	0.12861	✓
4	0.12118	1475.21062	0.00061	1.00182	1480.31694	0.00066	3.51248	0.60954	0.1296	✓
5	0.85603	1452.09173	0.00055	1.16333	1521.03222	0.00472	3.55188	0.5459	0.1658	✓
6	1.2001	1105.00065	0.00053	1.2961	1167.71698	0.00872	3.43308	0.50642	0.18917	✓
7	0.16312	1107.82211	0.00053	1.0087	1113.2199	0.0006	3.37024	0.60735	0.12728	✓
14	0.00038	1271.95599	0.00052	0.99253	1273.68928	0.00053	3.43585	0.61608	0.12741	✓
15	0.14052	1071.00125	0.00045	1.00504	1075.33804	0.00048	3.35281	0.60959	0.12546	✓
16	0.15915	1548.85809	0.0008	1.00716	1556.51733	0.00079	3.53887	0.60829	0.12795	✓
17	0.85039	1417.12248	0.00052	1.16034	1484.26737	0.00471	3.53959	0.54702	0.1689	✓
18	0.27712	1389.63518	0.00086	1.01353	1408.25068	0.00151	3.49078	0.60554	0.13761	✓

**Table 5:** Aalto university entrance hall links parameters of the $\alpha$–$\mu$ distribution.

TX	$d\;\left( \text {m}\right)$	$\alpha$	$\mu$	$\beta$	$KL_{\alpha{\text{--}}\mu }$	KS-test	*N*	LoS
1	4.35	3.1917	8.52562	14.31974	0.01114	✓	128	✓
1	4.35	3.20544	7.80577	14.0391	0.00896	✓	135	✓
1	4.35	3.06577	7.76737	14.09747	0.00865	✓	140	✓
1	4.35	3.15609	7.19428	14.21386	0.00979	✓	131	✓
2	9.82	3.12598	3.76144	11.35664	0.00723	✓	157	✓
3	3.3	3.0019	0.65597	10.77154	0.00189	✓	90	✗
4	17.01	8.54867	0.23377	8.49874	0.09188	✓	72	✗
5	11.24	2.59405	0.72646	7.10357	0.00539	✓	93	✗
5	11.24	2.64421	0.70618	7.20076	0.00616	✓	98	✓
6	23.31	2.47864	0.77571	7.27172	0.0036	✓	100	✗
6	23.31	2.45479	0.78466	6.8847	0.00494	✓	92	✓
7	28.71	2.47576	0.77262	3.65343	0.0137	✓	96	✗
7	28.71	2.31954	0.84535	3.73655	0.01019	✓	106	✓
8	20.16	–	–	–	–	✗	–	✗
9	30.65	2.29103	0.84712	6.34626	0.00209	✓	91	✗
9	30.65	2.2626	0.86197	6.34263	0.00299	✓	96	✓
10	47.44	3.35231	0.76845	3.72899	0.01425	✓	79	✗
10	47.44	3.23709	0.78004	4.1562	0.01553	✓	91	✓
10	47.44	3.26346	0.74307	4.29658	0.01143	✓	98	✓
11	12.59	–	–	–	–	✗	–	✗
12	10.19	3.25434	0.72028	3.05357	0.00943	✓	66	✗
12	10.19	3.33598	0.67266	3.24761	0.0066	✓	71	✓
12	10.19	3.36397	0.82513	2.6281	0.05727	✓	99	✓

**Table 6:** Aalto university entrance hall links parameters of the Rice, Nakagami-m and Lognormal distributions.

TX	*K*	$\Omega _{R}$	$KL_{R}$	*m*	$\Omega _{N}$	$KL_{N}$	$\mu _L$	$\sigma _L$	$KL_L$	KS-test
1	41.33417	202.47712	0.01289	21.32192	203.64305	0.02639	2.65224	0.10913	0.13377	✓
1	37.99915	194.39711	0.010615	19.65283	195.61089	0.02705	2.63161	0.11376	0.14845	✓
1	34.50664	195.94360	0.01045	17.90604	197.29479	0.02273	2.63532	0.11929	0.14441	✓
1	33.76636	199.01097	0.0121	17.53703	200.40682	0.02825	2.64297	0.12056	0.15932	✓
2	16.3451	125.28961	0.00919	8.82839	127.04055	0.04571	2.40806	0.17157	0.33576	✓
3	1.00556	102.30657	0.00806	1.21177	107.57785	0.04164	2.23324	0.53145	0.65784	✓
4	2.37218	54.60506	0.2489	1.7882	57.18999	0.34211	1.94932	0.43335	1.00137	✓
5	0.58391	46.30461	0.01209	1.07082	47.8604	0.03551	1.80937	0.58324	0.82824	✓
5	0.59205	47.35078	0.0151	1.07055	48.97989	0.04133	1.82183	0.5804	0.86	✓
6	0.5672	49.06032	0.00655	1.07073	50.6571	0.02444	1.83764	0.58203	0.80134	✓
6	0.55011	44.10787	0.00796	1.06747	45.47532	0.02547	1.78247	0.58408	0.77952	✓
7	0.54219	12.40027	0.02283	1.06435	12.77877	0.06195	1.14749	0.58472	1.60219	✓
7	0.49299	13.19363	0.01201	1.05795	13.55229	0.03674	1.17574	0.58593	1.52088	✓
8	–	–	–	–	–	–	–	–	–	✓
9	0.42231	38.30319	0.00298	1.03962	39.18869	0.01114	1.70433	0.5942	0.74334	✓
9	0.40998	38.39474	0.00347	1.03836	39.23919	0.01087	1.70398	0.59398	0.71621	✓
10	2.22322	12.22932	0.02219	1.7749	12.94193	0.23203	1.21198	0.41462	2.26706	✓
10	2.06452	15.2191	0.02009	1.69935	16.11801	0.2126	1.31792	0.42488	2.23007	✓
10	1.91742	16.2035	0.0136	1.62373	17.17584	0.18261	1.34607	0.43789	2.14394	✓
11	–	–	–	–	–	–	–	–	–	✓
12	1.78034	8.16321	0.02394	1.55786	8.65237	0.19349	1.00059	0.45028	2.15852	✓
12	1.65987	9.17678	0.02174	1.49785	9.72938	0.19358	1.05619	0.46195	2.23229	✓
12	2.55675	6.11535	0.08426	1.93913	6.45691	0.52751	0.87133	0.39511	4.39649	✓

### Fitting of $\alpha$–$\mu$ to the channel gain measurements

**Figure 3:**
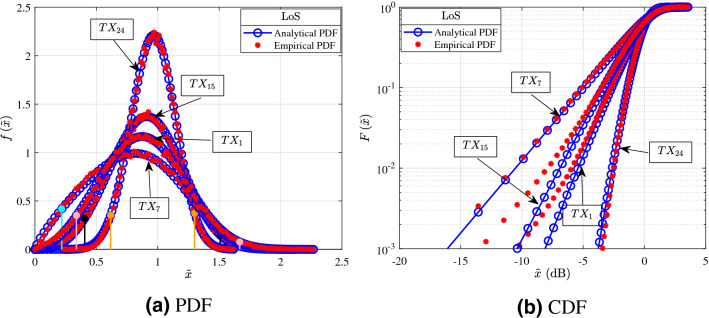
Fitting of PDF and CDF analytical expressions to the empirical channel gain data of some indicative LoS links (1, 7, 15, and 24) for the shopping mall measurements.

**Figure 4:**
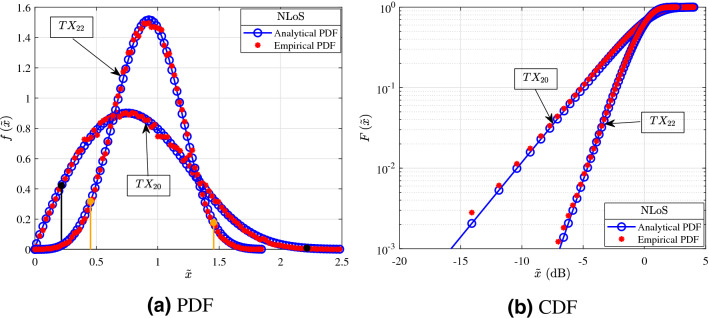
Fitting of PDF and CDF analytical expressions to the empirical channel gain data of the NLoS links (20 and 22) for the shopping mall measurements.

**Figure 5:**
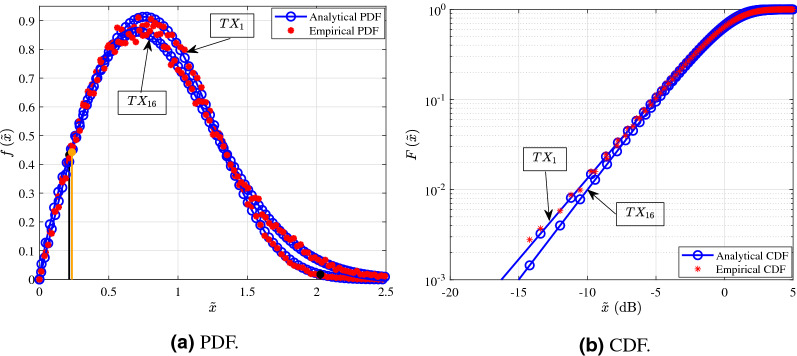
Fitting of PDF and CDF analytical expressions to the empirical channel gain data of some indicative links (1 and 16) for the airport check in area.

**Figure 6:**
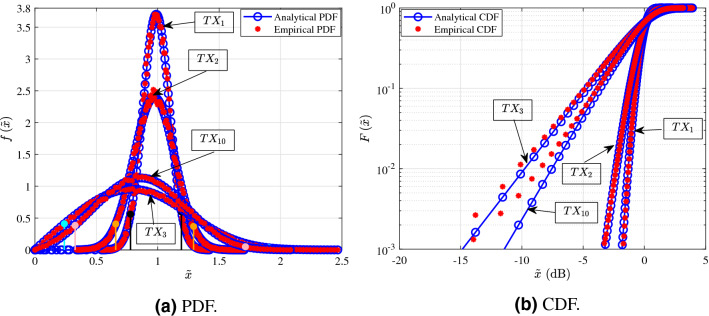
Fitting of PDF and CDF analytical expressions to the empirical channel gain data measurements of some indicative links (1, 2, 3 and 10) of the entrance hall of Aalto university.

The Figs. 3, 4, 5 and 6 shown in this section serve as illustrative examples of the fit achieved by the $\alpha$–$\mu$ distribution to the empirical channel gain PDFs and CDFs of both LoS and NLoS links of the three presented measurement scenarios. In more detail, the curves shown in Figs. 3, 4, 5 and 6 illustrate the PDF and CDF of ${{\tilde{x}}=x/\beta }$, i.e. *x* is normalized with respect to parameter $\beta$.

Figure 3 depicts indicative examples for the PDF and CDF of the channel according to measurements of LoS links that were conducted in the shopping mall. In more detail, Fig. 3a,b present the analytical and empirical PDF and CDF, respectively. From Fig. 1a, the links 1 and 24 were chosen, because they have relatively short transmission distances, whereas the links 7 and 15 were selected, due to the fact that they have relatively large transmission distances. Of note the transmission distance of links 1, 7, 15 and 24 are respectively 5.1, 65.2, 25.03, and $3.1\,{\text {m}}$. Moreover, in Fig. 3a the cyan, pink, black and orange vertical lines indicate the $95\%$ confidence interval of the median for the links 7, 1, 15, and 24, respectively. From Fig. 3a, it is observed that as the transmission distance increases, both the samples median as well as the range of their $95\%$ confidence interval decreases. This is due to the fact that as the distance increases, the number of reflected paths that carry a measurable amount of power decreases. Meanwhile, from this figure, it becomes evident that the $\alpha$–$\mu$ distribution provides an excellent fit with the experimental results. Furthermore, Fig. 3b provides an illustration that verifies the good fit results that are achieved by employing the KS test.

In Fig. 4 the PDF and CDF of the channel according to the measurements of the NLoS links that were conducted in the shopping mall are illustrated. More specifically, Fig. 4a,b present the analytical and empirical PDF and CDF, respectively. Also, links 20 and 22 have distances of 33.13 and $26.98\,{\text {m}}$, respectively. Additionally, the orange and black vertical lines indicate the $95\%$ confidence interval of the median for the links 22 and 20, respectively. From Fig. 4a it is observed that as the transmission distance increases both the samples median and their $95\%$ confidence interval range decreases. This is due to the fact that as the distance increases, the number of reflected paths that carry a significant amount of power decreases. Additionally the number of the reflected paths capable of being detected by the RX are further reduced by the obstacles, which absorb and scatter them. By taking this into account, it should be noted that link 20 has greater median range compared to link 22, because 22 is obstructed by a pillar made of solid material, whereas 22 is obstructed by a glass escalator. Meanwhile, from Fig. 4 it is evident that the $\alpha$–$\mu$ distribution provides an excellent fit for the NLoS links. Furthermore, Fig. 4b provides an illustrative example to verify the good fit that the KS test yields.

In Fig. 5 indicative examples for the PDF and CDF of the channel according to measurements of LoS and NLoS links that were conducted in the airport are presented. In more detail, Fig. 5a,b present the analytical and empirical PDF and CDF, respectively. In Fig. 5a, the LoS link 1 has a transmission distance of 5.1 m, while link 16 was the only NLoS link measured in this scenario and has a transmission distance of 20.09 m. In this figure it should be noted that the orange and black vertical lines indicate the $95\%$ confidence interval of the median for the links 16 and 1, respectively. From Fig. 5a, it is observed that as the transmission distance increases, both the samples median as well as the range of their $95\%$ confidence interval decreases. This is justified by the fact that as the transmission distance increases, the number of the reflected paths that carry a significant amount of power decreases. Meanwhile, Fig. 5 shows that the $\alpha$–$\mu$ distribution provides an excellent fit with the experimental results. Furthermore, by employing Fig. 5b it can be verified that the KS test yields a good fit.

In Fig. 6 indicative examples for the PDF and CDF of the channel according to measurements of LoS and NLoS links that were conducted in an entrance hall of the Aalto university campus are shown. Figure 6a,b present the analytical and empirical PDF and CDF, respectively. In Fig. 6a, the NLoS links 3, and 10 were selected, because they have transmission distances of 3.3, and 47.44 m, respectively. Additionally, in this figure the unique for this scenario LoS links 1 and 2 are presented. Moreover, it should be noted that the black, orange, cyan and pink vertical lines indicate the $95\%$ confidence interval of the median for the links 1, 2, 3, and 10, respectively. From Fig. 6a, it is observed that for the NLoS links as the transmission distance increases, both the samples median as well as the range of their $95\%$ confidence interval decreases. This is justified by the fact that, the increase of the transmission distance reduces significantly the number of the reflected paths that carry a measurable amount of power. Furthermore, the same observation about the median applies also for the LoS links 1 and 2. Meanwhile, Fig. 6 illustrates that the $\alpha$–$\mu$ distribution provides an excellent fit with the experimental results. Additionally, Fig. 6b serves as an illustrative example to verify the good fit that is achieved by means of the KS test.

The Tables 1, 3 and 5 show the parameters of the $\alpha$–$\mu$ distribution that were found to fit the empirical measurement channel data for the measurements sites of the shopping mall, airport check in area and Aalto campus entrance hall. In all the presented scenarios the $\alpha$–$\mu$ distribution parameters provide an adequate fit to the empirical data, by passing the KS-test, while yielding also low values for the KL test. The only exception are links 8 and 11 presented in Table 5, because for those links no paths were detected by the receiver. By observing the values of the parameter $\alpha$ in the three presented scenarios it can be seen that for the majority of the measured links, $\alpha \in \left[ 2-3\right]$. An exception is made by the links 4, 5, 7, 14 of the shopping mall measurement scenario, where $\alpha \in \left[ 4-6.5\right]$ and for link 4 of the Aalto entrance hall measurements where $\alpha =8.54867$. Furthermore, by observing the values of the parameter $\mu$ in the three scenarios it can be seen that $\mu \in \left[ 0.23-8.5\right]$. Moreover, it can be noticed that the NLoS links in all the three scenarios have $\mu \le 1$.

## Discussion

According to the literature, the THz channel modeling up until now was performed by employing fading distributions such as, Nakagami-m, Rayleigh, Rice, Weibull and mixture of Gamma distributions^4,15–18^. In this work, the suitability of modeling the THz small-scale fading by means of the $\alpha$–$\mu$ distribution is examined. Towards this direction despite the paramount importance of pathloss modeling, in this work, we normalized the path gain measurements of each link as in Eq. (2). This was administered in order to eliminate the effect of the deterministic pathloss and retain only the small-scale fading characteristics of the measured channels. Moreover, the deterministic THz pathloss and its dependency on the operating frequency, transmission distance, relative humidity, air temperature, and pressure has been studied extensively in previous works^3,10–13,19–21,23,25,43^.

It is observed that, $\alpha$–$\mu$ accomplishes a good fit to the channel gain measurements of all the links in the shopping mall, airport check in area and Aalto university entrance hall scenarios. In more detail, the goodness of fit of $\alpha$–$\mu$ is compared to that of the Nakagami-m, Rice, Rayleigh and lognormal fading distributions and it is evaluated by means of the KS and KL tests. For the shopping mall scenario, the empirical PDF of the channel gain measurements of the links 4, 5, 7, 12, 14, 16, 18, 19 and 21 is more accurately fitted by the $\alpha$–$\mu$ distribution, when compared to Nakagami-m and Rice. This is verified by the values of ${\text {KL}}_{\alpha{\text{-}}\mu }$, ${\text {KL}}_{N}$ and $KL_{R}$, where by observing Tables 1 and 2, ${{\text {KL}}_{\alpha{\text{-}}\mu }}$ has the lowest value. Furthermore, by observing the KL values of Tables 3 and 4, $\alpha$–$\mu$ accomplishes a better fit to the empirical channel gain PDF of link 1 of the airport check in area. For the Aalto university entrance hall link 4 from Tables 5 and 6 based on the KL value it is observed that $\alpha$–$\mu$ yields a better fit to the empirical PDF compared to Rice and Nakagami-m. Meanwhile, by observing the KL values of Tables 1, 2, 3, 4, 5 and 6 it can be conducted that Nakagami-m performs the worst in terms of fitting to the empirical channel gain PDFs, when compared to $\alpha$–$\mu$ and Rice distributions. Meanwhile, according to the KL test values the lognormal distribution performed the worst in terms of fitting to the empirical channel gain distributions, when compared to $\alpha$–$\mu$, Rice and Nakagami-m. Finally, it should be noted that, the Rayleigh distribution in all the measurement scenarios and for all the links did not yield an adequate fit even in terms of the KS test.

## Methods

### Preprocessing of the measurement data

The channel describing a wireless RF link is expressed in terms of a product of two coefficients, one deterministic and one stochastic. The deterministic part describes the large-scale fading effects of the propagation, i.e the pathloss. In more detail, the large-scale fading describes time-invariant phenomena of the signal propagation, whose effect remains constant during the signal propagation. Meanwhile, the stochastic channel coefficient describes the small-scale fading characteristics of the channel, which are time and frequency dependent. The small-scale fading behavior is of special importance because it can lead to unpredicted deep fades to the received signal power. In this sense in order to perform small-scale fading characterization of the channel one should eliminate the deterministic channel coefficient of pathloss. The channel sounding performed in the shopping mall, airport and Aalto university entrance hall measurements environments provides power angular delay profiles (PADPs) for each of their TX–RX links. In more detail, the PADPs of each link are given as a set of propagation paths

1 $$\begin{aligned} {\text {PADP}}\left( \phi ,t\right) = \sum _{i=1}^{I} G P_i \delta \left( \phi -\phi _i\right) \delta \left( t-t_i\right) , \end{aligned}$$

where $\phi _i$, $P_i$ and $t_i$ stand for the azimuth angle at the RX, the propagation delay gain and time of the *i*th propagation path, respectively. The parameter *G*, known as the broadside angle, denotes the combined gains of the TX and RX antennas, while *I* and $\delta \left( \cdot \right)$ are the Dirac delta function and the total number of multipath components of a link, respectively. Then, in order to eliminate the deterministic phenomenon of pathloss, the link path gain measurements by employing Eq. (1) to each link, are normalized to unity as

2 $$\begin{aligned} \zeta _i^2=\frac{P_i}{{{\overline{p}}}}, \end{aligned}$$

where

3 $$\begin{aligned} {\overline{p}}=\frac{\sum _{i=1}^{I} P_i}{I}. \end{aligned}$$

### Generation of different channel realizations from a single measurement

In the THz band the wavelength of the transmitted electromagnetic waves is much smaller compared to the size of obstacles laid in the transmission path. This reduces the ability of the signals to diffract around obstacles leading to received signal power attenuation of even ${40\;{\text {dB}}}$^25,37^. Furthermore, the severe propagation losses due to the water vapor density and the temperature of the atmospheric medium make the THz wireless transmissions to heavily rely on the LoS component of the channel^12,19,43^. In this sense the THz band has non-rich multipath environments. However, still there are surfaces that can act scatters in the THz band^12,13,17,21^. This leads to some reflected multipath components with a significant amount of power that arrive to the RX from NLoS directions. Despite this fact, still the number of measured multipath components is not sufficient enough to perform small-scale fading statistics analysis for a THz channel. To tackle this limitation, one can generate different realizations of the transfer function by changing the phases of the multipath components^18,44^. The phases are assumed to be stochastic following a uniform distribution in the interval ${\left( 0,2{\text { }\pi } \right) }$. Then, the channel coefficient of a single-input–single-output (SISO) system can be obtained as^44^

4 $$\begin{aligned} h=\sum _{i=1} \zeta _i \exp \left( -j2\pi f t_i\right) \exp (j \psi _i), \end{aligned}$$

where ${\psi _i\ \sim U\left( 0,2\text { }\pi \right) }$ represents the random phase of the *i*th multipath component. Moreover, by assuming that the amplitude of the channel coefficients does not change dramatically among the progressing $t_i$, i.e. the channel can be considered as flat-fading then, ${t_i=0}$^44^. Also, the term ${U\left( \cdot ,\cdot \right) }$ is the uniform distribution operator^30^.

### The $\alpha$–$\mu$ distribution

The $\alpha$–$\mu$ distribution has been widely used in describing the small-scale fading statistics of RF wireless channels. It offers not only mathematical tractability, but also encapsulates as special cases important distributions of statistical analysis^38,45^. By setting the parameters $\alpha$ and $\mu$ to appropriate values one can obtain distributions such as Nakagami-m, Gamma, Rayleigh, Weibull, exponential and one-sided Gaussian. The PDF and CDF of $\alpha$–$\mu$ are expressed as^38^

5 $$\begin{aligned} f\left( x\right)&=\frac{\alpha \mu ^{\mu }\left( \frac{x}{\beta }\right) ^{\alpha \mu -1}\exp \left( -\mu \left( \frac{x}{\beta }\right) ^{\alpha }\right) }{\beta \Gamma \left( \mu \right) }, \end{aligned}$$

6 $$\begin{aligned} F\left( x\right)&=1-\frac{\Gamma \left( \mu ,\left( \frac{x}{\beta }\right) ^{\alpha }\mu \right) }{\Gamma \left( \mu \right) }, \end{aligned}$$

where, $\beta$ and $\mu$ are obtained as^38^

7 $$\begin{aligned} \beta&=\root \alpha \of {E\left( X^{\alpha }\right) }, \end{aligned}$$

8 $$\begin{aligned} \mu&=\frac{E^2\left( X^{\alpha }\right) }{V\left( X^{\alpha }\right) }. \end{aligned}$$

The parameter $\alpha >0$ expresses the non-linearity of the received signal envelope due to the propagation environment, while the parameter $\mu >0$ stands for the number of the multipath components of the received signal^38^. The non-integer values of $\mu$ may be justified as non-zero correlation among the in-phase and quadrature parts of the multipath component, non-zero correlation between different clusters of multipath components, or non-Gaussianity of the in-phase and quadrature components of the fading signal^38^. Moreover, *X* is a random variable (r.v.) following the $\alpha$–$\mu$ distribution, meanwhile $\Gamma \left( \cdot \right)$ and $\Gamma \left( \cdot ,\cdot \right)$ stand for the gamma function and the upper incomplete gamma function, respectively^46^, Eqs. (8.310.1) and (8.350.2).

### The Rice, Nakagami-m, Rayleigh and Lognormal distributions

The Rice, Rayleigh, Nakagami-m and lognormal distributions are widely used in modeling the fading statistics of RF wireless channels, while they have been also used to model the small-scale fading of THz wireless channel measurements^15,17,18^. The PDF and CDF of the Rice distribution is expressed as^30^, Eq. (3.37)]

9 $$\begin{aligned} f_{R}\left( x\right)&= 2 x \frac{K+1}{\Omega _R} \exp \left( -K-\frac{\left( K+1\right) x^2}{\Omega _R}\right) I_o\left( 2x \sqrt{\frac{K \left( K+1\right) }{\Omega _R}}\right) , \end{aligned}$$

10 $$\begin{aligned} F_{R}\left( x\right)&= Q_1\left( \sqrt{2}K,0\right) -Q_1\left( \sqrt{2}K, \frac{x}{\frac{\sqrt{2}}{2} \sqrt{\frac{\Omega _R}{K+1}}}\right) , \end{aligned}$$

where $Q_1\left( \cdot ,\cdot \right)$ is the fisrt order Marcum-Q function^47^. The parameter *K* represents the ratio of power of the LoS signal component to the other NLoS signal components, while $\Omega _R$ stands for the average received signal power. The PDF and CDF of the Nakagami-m distribution are obtained as^30^, Eq. (3.38)]

11 $$\begin{aligned} f_m\left( x\right)&=\frac{2 \exp \left( -\frac{\mu x^2}{\Omega _N }\right) \left( \frac{\mu }{\Omega _N}\right) ^{\mu } x^{-1+2 \mu }}{\Gamma \left( \mu \right) }, \end{aligned}$$

12 $$\begin{aligned} F_m\left( x\right)&=1-\frac{\Gamma \left( m,\frac{m x^2}{\Omega _N }\right) }{\Gamma \left( m\right) }, \end{aligned}$$

where the parameters *m* and $\Omega _N$ are the fading parameter and the average received signal power, respectively. The PDF and CDF of the Rayleigh distribution is expressed as^30^, Eq. (3.32)]

13 $$\begin{aligned} f_{rl}\left( x\right)&=\frac{x}{\sigma ^2}\exp \left( -\frac{x^2}{2 \sigma ^2}\right) , \end{aligned}$$

14 $$\begin{aligned} F_{rl}\left( x\right)&=1-\exp \left( -\frac{x^2}{2 \sigma ^2}\right) , \end{aligned}$$

where $\sigma$ is the variance. The PDF and CDF of the lognormal distribution are obtained as^39^ Eqs. (2.1) and (2.4)

15 $$\begin{aligned} f_L\left( x\right)&=\frac{\exp \left( -\frac{\left( -\mu _L+ln\left( x\right) \right) ^2}{2 \sigma _L^2}\right) }{\sqrt{2 \pi }x \sigma _L}, \end{aligned}$$

16 $$\begin{aligned} F_L\left( x\right)&=\frac{1}{2}\text {Erfc}\left( \frac{\mu _L-ln\left( x\right) }{\sqrt{2}\sigma _L}\right) , \end{aligned}$$

where $\mu _L$ and $\sigma _L$ stand for the mean and standard deviation, respectively.

### Evaluation of the fitting

To evaluate the fitting of the Rice, Nakagami-m and $\alpha$–$\mu$ fading distributions to the empirical distribution of the channel gain of each link two goodness of fit methods were employed. Namely, the Kolmogorov–Smirnov and Kullback–Leibler divergence tests.

### Kolmogorov–Smirnov goodness of fit test

The Kolmogorov–Smirnov goodness of fit test is defined as^40^

17 $$\begin{aligned} max\left( \left| F_{emp}\left( x\right) -F\left( x\right) \right| \right) \le \sqrt{-\frac{1}{2N} ln{\left( \frac{A}{2}\right) }}, \end{aligned}$$

where $F_{emp}\left( x\right)$ and *N* stand for the empirical values of the channel gain CDF of the examined link and the number of discrete samples of $F_{emp}\left( x\right)$, respectively. The parameter $F\left( \cdot \right)$ denotes the analytical CDF of the examined distribution, while $A=5\%$ is the selected significance level.

### Kullback–Leibler divergence test

The Kullback–Leibler divergence test is defined as the distance between the empirical PDF $f_{emp}\left( x\right)$ and the analytical PDF $f\left( x\right)$ of the examined distribution^41^

18 $$\begin{aligned} KL=-\sum _{x\in \chi } f_{emp}\left( x\right) ln \left( \frac{f\left( x\right) }{f_{emp}\left( x\right) }\right) . \end{aligned}$$

The closer the value of Eq. (18) to 0 the better is the fit of the analytical fading distribution to the empirical channel gain distribution.

## Data Availability

The data are owned by Nokia Bell-Labs and Aalto University. Any researcher affiliated to one of the ARIADNE project partners is allowed to access and use the shared data for research purposes. The shared data must however not be made accessible to any person not affiliated with any ARIADNE project partner.
